# Do patient characteristics matter when calculating sample size for eczema clinical trials?

**DOI:** 10.1002/ski2.42

**Published:** 2021-05-19

**Authors:** L. Howells, S. Gran, J. R. Chalmers, B. Stuart, M. Santer, L. Bradshaw, D. M. Gaunt, M. J. Ridd, L. A. A. Gerbens, P. I. Spuls, C. Huang, N. A. Francis, K. S. Thomas

**Affiliations:** ^1^ Centre of Evidence Based Dermatology School of Medicine University of Nottingham King's Meadow Campus Nottingham UK; ^2^ Primary Care Research Centre School of Primary Care Population Sciences and Medical Education University of Southampton Southampton UK; ^3^ Nottingham Clinical Trials Unit University of Nottingham Nottingham UK; ^4^ Bristol Medical School: Population Health Sciences University of Bristol Bristol UK; ^5^ Department of Dermatology Amsterdam Public Health Infection and Immunity Amsterdam UMC University of Amsterdam Amsterdam the Netherlands; ^6^ Hull York Medical School University of Hull Hull UK; ^7^ Division of Population Medicine Cardiff University School of Medicine Cardiff UK

## Abstract

**Background:**

The Patient‐Oriented Eczema Measure (POEM) is the core outcome instrument recommended for measuring patient‐reported atopic eczema symptoms in clinical trials. To ensure that the statistical significance of clinical trial results is meaningful, trials are often designed by specifying the target difference in the primary outcome as part of the sample size calculation. One method used to specify the target difference is a score that corresponds to a standardized effect size.

**Objectives:**

to assess how the standardized effect size of POEM scores vary across age, gender, ethnicity and disease severity.

**Methods:**

This study combined data from five UK‐based randomized clinical trials of eczema treatments in order to assess differences in self‐reported eczema symptoms (POEM) corresponding to a standardized effect size (0.5 SD of baseline POEM scores) across age, gender, ethnicity and disease severity.

**Results:**

POEM scores corresponding to 0.5 SD_(baseline)_ were remarkably consistent across participants of varying ages, gender, ethnicity and disease severity from datasets of five UK trials in children (range 2.99–3.45).

**Conclusions:**

This study provides information that can support those designing clinical trials to determine their sample size and can aid individuals interpreting trial results. Further exploration of differences in populations beyond the United Kingdom is needed.

## Graphical abstract

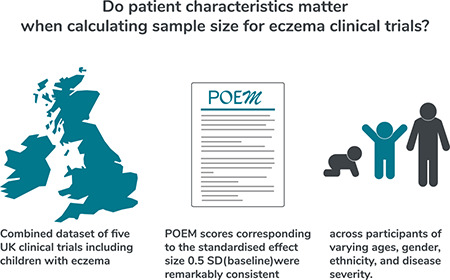

1

**WHAT IS ALREADY KNOWN ABOUT THIS TOPIC?**

- Little is known about how participant characteristics might influence the difference that is important to detect when calculating sample size requirements for clinical trials.

**WHAT DOES THIS STUDY ADD?**

- Using the combined dataset across UK clinical trials including children with eczema, POEM scores corresponding to the standardized effect size 0.5 SD_(baseline)_ was remarkably consistent across participants of varying ages, gender, ethnicity and disease severity.
- This provides reassurance for those designing or interpreting the results of eczema clinical trials that have used POEM as their primary outcome that the specified difference can be consistent in a variety of eczema populations and recruitment settings.

## 2 INTRODUCTION

The Patient‐Oriented Eczema Measure (POEM) is a 7‐question patient‐reported measure of atopic eczema severity assessed over the previous week and scored 0 to 28.^1^ POEM is recommended for inclusion in all eczema clinical trials by the Harmonising Outcome Measures in Eczema (HOME) initiative.^2^ When designing clinical trials with POEM as a primary outcome, the target difference in the primary outcome needs to be stated as part of the sample size calculation.^3^ Cook et al. have identified methods that can be used to assess whether the target difference specified is important and/or realistic.^3^

One approach to specifying a target difference is to use the minimally important change (MIC), defined as ‘the smallest change in score in the construct to be measured which patients perceive as important’.^4^ *Anchor‐based methods* to calculate the MIC of an instrument look how change on the instrument corresponds to change on a different instrument (the ‘anchor’).

Another method commonly used to specify the difference to be detected in sample size calculations is a *distribution‐based approach* based on the standardized effect size.^3^ The effect size is the change in scores between baseline and post‐treatment divided by the standard deviation of baseline scores.^5^, ^6^ It has been suggested the difference to be detected in clinical trials, based on a medium effect size, could be specified as the score that corresponds to 0.5 SD_(baseline)._ This is the method that is used within this study. Some have suggested this provides a threshold of detection, which is conceptually related to minimally detectable change.^4^ Essentially, this means eczema clinical trials should not design their studies to detect a target difference smaller than the score on POEM which corresponds to 0.5 SD_(baseline)_ as it is unlikely to be considered a meaningful difference in POEM scores.

We have explored different anchor‐ and distribution‐based methods in a previous article.^7^ Recommendations for interpreting changes in POEM scores based on three published studies using both anchor‐ and distribution‐based approaches suggest the following: ≤2, unlikely to be a change beyond measurement error; 2.1–2.9, a small change detected that is likely to be beyond measurement error but may not be clinically important; 3–3.9, probably a clinically important change; ≥4, very likely to be a clinically important change.^7^, ^8^, ^9^ However, participant characteristics could potentially influence what difference would be important to specify in a sample size calculation, thus potentially limiting the generalizability of these recommendations.^10^ The aim of this study was to assess how the POEM scores that correspond to the standardized effect size (0.5 SD) are influenced by age, gender, ethnicity and disease severity in these studies.

## 3 METHODS

Secondary analysis was conducted on datasets from five clinical trials including children from the United Kingdom. Data sets were chosen due to availability, so may not be inclusive of all eczema trials within the United Kingdom. As this study made secondary use of existing trial datasets, further ethics approval was not required, and this was confirmed by the University of Nottingham's Faculty of Medicine & Health Sciences Research Ethics Committee (Ref: 258‐1712). The protocol was prospectively registered on the CEBD protocol registration portal: https://www.nottingham.ac.uk/research/groups/cebd/resources/protocol‐registration.aspx.

POEM measures patient‐reported frequency of itch, sleep disturbance, bleeding, weeping/oozing, cracking, and flaking and dryness/roughness over the past week. Each item is weighted equally and scored as 0 (no days), 1 (1–2 days), 2 (3–4 days), 3 (5–6 days) or 4 (every day). Age, gender and ethnicity variables in each dataset were transformed into a priori defined categories (Table 1).

**TABLE 1:** Summary of datasets used

	CLOTHES	SWET	BATHE	COMET	CREAM
	Clothes for the relief of Eczema trial^11^	Softened Water Eczema Trial^12^	Bath additives for the Treatment of cHildhood Eczema trial^13^	Choice of Moisturiser for Eczema Treatment trial^14^	ChildRen with Eczema Antibiotic Management trial^15^
Trial registration number	ISRCTN: 77261365	ISRCTN: 71423189	ISRCTN: 84102309	ISRCTN: 21828118	ISRCTN: 96705420
Population trial aimed to recruit from	Moderate—severe eczema	Moderate—severe eczema	All severities of eczema (except very mild)	All severities of eczema	Clinically infected eczema
Setting of recruitment	Secondary care and self‐referral	Secondary care, primary care and self‐referral	Primary care	Primary care	Primary care
Location of recruitment	UK	UK	UK	UK	UK
*N*	300	336	482	196	112
Age *N* (%)					
0–2	125 (42)	131 (39)	191 (40)	170 (87)	65 (58)
3–7	114 (38)	136 (40)	221 (46)	26 (13)	47 (42)
8–17	61 (20)	69 (21)	70 (14)	0	0
Ethnicity *N* (%)					
Not white	63 (21)	74 (22)	75 (16)	29 (15)	19 (17)
White	237 (79)	260 (77)	397 (82)	155 (79)	91 (81)
Missing data	0	2 (1)	10 (2)	12 (6)	2 (2)
Female *N* (%)	126 (42)	143 (43)	244 (51)	85 (43)	51 (46)

Baseline POEM scores and patient characteristic variables from each trial were combined in one dataset in STATA Version 14. To explore the impact of disease severity, we calculated the overall mean, standard deviation and 0.5 SD of baseline POEM for each trial separately (as each trial recruited participants with different eczema severities). We also combined individual participant data to calculate the mean, standard deviation and 0.5 SD for overall sample and for each age, ethnicity and gender category.

## 4 RESULTS

Data from 1426 participants across five UK trials were combined. All five trials included children in the categories 0–2 years and 3–7 years. The CLOTHES, SWET and BATHE trials included some children in the 8–17 years category, however this was the least frequent age in all three trials. Gender was roughly equally distributed within all trials. Most participants across all trials were white (79%) (Table 1).

POEM scores corresponding to the standardized effect size from each of the five trials of children in the United Kingdom ranged from 2.68 to 2.95 (Table 2). When the individual participant data from these trials were combined and categorized according to age, ethnicity and gender, the POEM scores corresponding to the standardized effect size ranged from 2.99 to 3.45.

**TABLE 2:** UK trials including children: The POEM scores corresponding to 0.5 SD_(baseline)_ by trial, age, gender, ethnicity and baseline POEM scores (*N* 1426)

	*N*	0.5 SD_(baseline)_	Mean (SD)	Min. POEM score	Max. POEM score	Q_1,_ Q_3_ ^a^
Total sample	1426	3.37	13.23 (6.74)	0	28	8, 18
Severity of eczema in the population						
Moderate—severe eczema (CLOTHES)	300	2.68	16.95 (5.36)	4	28	13, 20
Moderate—severe eczema (SWET)	336	2.95	16.87 (5.90)	0	28	12.5, 22
All severities of eczema (BATHE)	428	2.88	9.77 (5.76)	0	28	6, 13
All severities of eczema (COMET)	196	2.94	8.80 (5.87)	0	28	4, 12
Clinically infected eczema (CREAM)	112	2.69	14.99 (5.38)	6	28	11, 18.5
Age						
0–2	682	3.25	12.46 (6.50)	0	28	8, 17
3–7	544	3.40	13.41 (6.80)	0	28	8, 18
8–17	200	3.44	15.39 (6.87)	0	28	9.5, 20.5
Ethnicity						
White	1140	3.45	14.36 (6.89)	0	28	8, 18
Not white	260	3.34	13.05 (6.68)	0	28	9, 20
Gender						
Male	777	3.43	13.55 (6.85)	0	28	8, 19
Female	649	3.30	12.85 (6.59)	0	28	8, 17

*Note:* Age, ethnicity and gender are the combined data from all five trials.

^a^ Q_1_ = lower quartile, Q_3_ = upper quartile.

## 5 DISCUSSION

The POEM score corresponding to the standardized effect size was remarkably consistent across participants of varying ages, gender, ethnicity and disease severity. Previous studies that have used both anchor‐ and distribution‐based methods to estimate the MIC found that anchor‐based methods suggested a larger difference would need to be detected than the standardized effect size approach.^7^, ^8^ Our findings are consistent with previous findings that a score less than 2 is unlikely to be a change beyond measurement error and support a stance that the specification of a target difference should be no lower than 2 when designing trials, as all 0.5 SD scores were above 2 points across the different participant characteristics. Previous research also suggests that changes in POEM scores less than 3 are unlikely to be clinically relevant. However, researchers and clinicians need to be cautious about relying on fixed values to interpret the importance of a change on the POEM and continue to consider the context within which they are using the POEM. A small improvement in many individuals could result in a large reduction in burden at a societal level.

It has been cautioned that the SD used to calculate the standardized effect size needs to reflect the SD for the population of interest for the results to be used appropriately.^3^, ^16^ This study should give reassurance that the participant characteristics of age, ethnicity, sex and disease severity do not appear to influence the SD.

Given that datasets were chosen based on availability, they may not be fully representative of eczema trials including children within the United Kingdom. There are also limits on how confidently the results from this study can be applied to wider populations beyond children in the United Kingdom, such as those nations with larger documented ethnic and economic disparities in health outcomes. Further exploration in different populations is warranted. We can compare our results with the 0.5 SD we have calculated for the trial of methotrexate versus azathioprine for severe atopic dermatitis (MAcAD) (Dutch trial register: NTR1916).^17^ MAcAD is a Dutch trial that included 43 adults with severe eczema. Twenty (46.5%) female and 38 (88.4%) reported white ethnicity. 0.5 SD from this sample was 2.32. This is slightly lower than that of the UK trials in children, but still relatively consistent with the results.^17^

There are characteristics that vary amongst individuals that may influence the target difference that should be specified in a clinical trial. Some examples include body surface area of involved skin, phenotype, and whether the POEM was completed by parent or the child themselves. We were limited to exploring variables that were collected in the trial datasets.

The POEM score corresponding to the standardized effect size does not appear to be influenced by age, gender, ethnicity or disease severity of the population, which provides useful information for those designing or interpreting trials with POEM as the primary outcome.

## CONFLICT OF INTEREST

L. Howells, J. R. Chalmers, K. S. Thomas, B. Stuart, L. A. A. Gerbens and P. I. Spuls are members of the HOME initiative. The Patient Oriented Eczema Measure (POEM) was developed at the Centre of Evidence Based Dermatology, University of Nottingham. D. M. Gaunt discloses personal consulting fees from Pfizer Inc; these are unrelated to this research. L. Howells currently acts as consultant for the University of Oxford on an educational grant funded by Pfizer, unrelated to the submitted work.

## Data Availability

To access the datasets used in this study please contact the chief investigator and/or corresponding author for each individual clinical trial (contact details can be found in the original trial publications which are referenced within this study).
